# Multivariate analysis of agronomic characteristics in some Egyptian barley landraces: a field-based quantitative study

**DOI:** 10.1186/s12870-025-07512-8

**Published:** 2025-11-14

**Authors:** Mohamed Abdelghany, Khamis I. Saad, Mohamed Dakrory, Khaled E. Amer

**Affiliations:** 1 Crop Science Department, Faculty of Agriculture, Damanhour University, Damanhour, 22516 Egypt; 2 Genetic Resources Research Department (GRRD), Field Crops Research Institute (FCRI), Agricultural Research Center (ARC), Giza, 12619 Egypt

**Keywords:** Principal component analysis, Phenotypic diversity and cluster analysis

## Abstract

**Background:**

Barley is a significant cereal crop in Egypt, with the potential to be used for its endurance against harsh environmental conditions, particularly in arid and semi-arid regions. The current study was conducted to assess the genetic diversity and relationships among agronomic traits of 81 Egyptian barley landraces using multivariate statistical tools like principal component analysis, cluster analysis, path analysis, and correlation analysis. The parameters recorded were days to heading, days to maturity, plant height, grain yield, spikes per square meter, grains per spike, and thousand-grain weight.

**Results:**

The Pearson correlation heatmap showed strong relationships between the agronomic characteristics assessed in 81 barley landraces. The number of spikes per square meter and grain yield showed the largest favorable relationships (*r* = 0.77). The circular dendrogram revealed four major clusters with distinct branch groupings related to the 81 landraces. The hierarchical clustering of the traits resulted in the formation of two large clusters. Cluster I includes days to heading and maturity. The remaining five traits are separated into two smaller groups and belong to cluster II. The first and second primary components account for 53.6% of the entire variation, with 31.1% and 22.5% of the total variation, respectively. Regarding the direct effects, grain yield was most positively impacted by the number of spikes per square meter. Grain yield was indirectly positively impacted by plant height and the number of spikes per square meter through the number of grains per spike characteristic.

**Conclusions:**

Multivariate analysis is employed in the revelation of interrelationships’ correlations of traits among Egyptian barley landraces. Number of spikes per square meter and number of grains per spike were the most correlated traits influencing grain yield and, therefore, should be prioritized in breeding schemes. The findings can be used to aid the selection of high-performance genotypes for yields improvement and tolerance to stressful environments.

**Supplementary Information:**

The online version contains supplementary material available at 10.1186/s12870-025-07512-8.

## Introduction

 One of the first crops that humans domesticated was barley (*Hordeum vulgare* L.). According to current genomic and archaeological data, barley is a mosaic crop that evolved from multiple populations; it is possible that domesticated barley didn’t originate from a single source [1]. In the Gramineae family’s Triticaceae, barley is a member of the genus Hordeum. One of the most significant cereal crops grown globally, barley can be found in various climates, including temperate, subtropical, desert, and semi-arid [2]. Barley is one of the most significant and cost-effective cereal crops, ranking fourth globally in terms of food crop production behind rice, wheat, and maize [3]. With a genome size of 4.79 billion letters of genetic code, double the size of the human genome, it is a diploid (2 *n* = 2x = 14) and one of the largest cereals [4]. The crop’s adaptability is well-known, and natural stands can be found in a greater variety of agroecologies, ranging from the arid Mediterranean to the Himalayas at elevations of up to 4,500 m [5]. Its primary uses are for feed (55–60%), malt brewing (30–40%), and food (the remaining percentage) [6]. It is also a significant supplier of raw materials for the brewing and malting sectors [7]. Additionally, barley is utilized in a wide range of sectors, including manufacturing baby food, energy drinks, vinegar, beer, and medicinal syrups [8].

Barley was first cultivated in Egypt between 5000 and 6000 BC, according to archeological data [9]. A full diet in ancient Egypt consisted of barley goods, especially bread and beer. Due to its health benefits, the need for agricultural growth, and the need to cut back on wheat imports, Egypt is actively looking into reintroducing barley as a 30% ingredient in bread manufacture. In the 2017–2018 crop year, 142.37 million metric tons of barley were produced worldwide [10]. In addition, barley production is predicted to drop to 140.6 million metric tons in the upcoming crop year [11]. Egypt’s barley production has grown over the last 20 years and reached 108,000 tons in 2019 [10], but has varied significantly in recent years. It is anticipated that the area and productivity will rise as a result of the new sustainable development plan regulations and the extension of reclaimed land [12]. According to Aly et al. [13], barley is an essential crop in Egypt and accounts for 76.9% of the recently reclaimed land. Egypt’s barley production was 90 thousand metric tons in 23/2024 and is predicted to continue rising in the coming years [12]. In addition to being used to make bread and other nutritious foods, barley can be used as animal feed [14]. Adding barley to wheat flour to make bread is one of the proposed ways to address Egypt’s wheat crisis [15].

All statistical techniques that concurrently examine several measurements on each person or item under study are called multivariate analysis. More specifically, multivariate analysis can be defined as any analysis that involves more than two variables [16]. One crucial tactic for classifying germplasm and examining the genetic interactions between genotypes is the application of multivariate techniques [17]. Numerous crop species have made extensive use of multivariate analysis of quantitative variables to predict genetic diversity [18]. Like most cereals, barley experienced genetic bottlenecks during agricultural improvement and domestication. Principal component analysis (PCA) has been utilized extensively for genotype grouping and variable reduction [19]. PCA provides dependable distribution patterns and removes redundancy from data sets [20]. PCA has emerged as a reliable multivariate statistical technique for dimensionality reduction in data, with the retention of crucial variation [21]. PCA enables one to identify dominant traits responsible for general phenotypic and genetic variation, assisting plant breeders to select the best genotypes efficiently [22]. A popular multivariate methodology for characterizing genetic diversity based on genotype similarities or differences is cluster analysis [21]. Cluster analysis is a generic technique in data analysis and machine learning for the discovery of concealed patterns within a dataset [23]. It is a generic unsupervised learning method with generic uses in fields as diverse as bioinformatics, image processing, market segmentation, and social network research [24]. Despite its great effectiveness, cluster analysis has a number of drawbacks, such as figuring out how many clusters to use, dealing with high-dimensional data, and handling overlapping or unbalanced clusters. Modern computational techniques, such as deep clustering and mixed algorithms, have attempted to overcome these drawbacks and increase clustering accuracy [25].

Despite the recognized worth of landraces in ensuring genetic diversity, there is little systematic agronomic and genetic evaluation of these resources in Egypt. Limited data for elaborate, trait-based information prevents the realization of the productive integration of landraces into yield improvement, resistance, and adaptability programs. Multivariate methodologies have to be applied to reveal trait interactions and genotype classification in a manner that facilitates improvement planning [22]. The present study aims to assess the genetic and phenotypic variation among 81 Egyptian barley landraces using multivariate statistical techniques. It seeks to identify key agronomic traits influencing variability and to classify landraces into distinct groups based on their performance, providing insights for breeding programs focused on improving yield, adaptation, and stress resistance.

## Materials and methods

### Plant materials

**Fig. 1:**
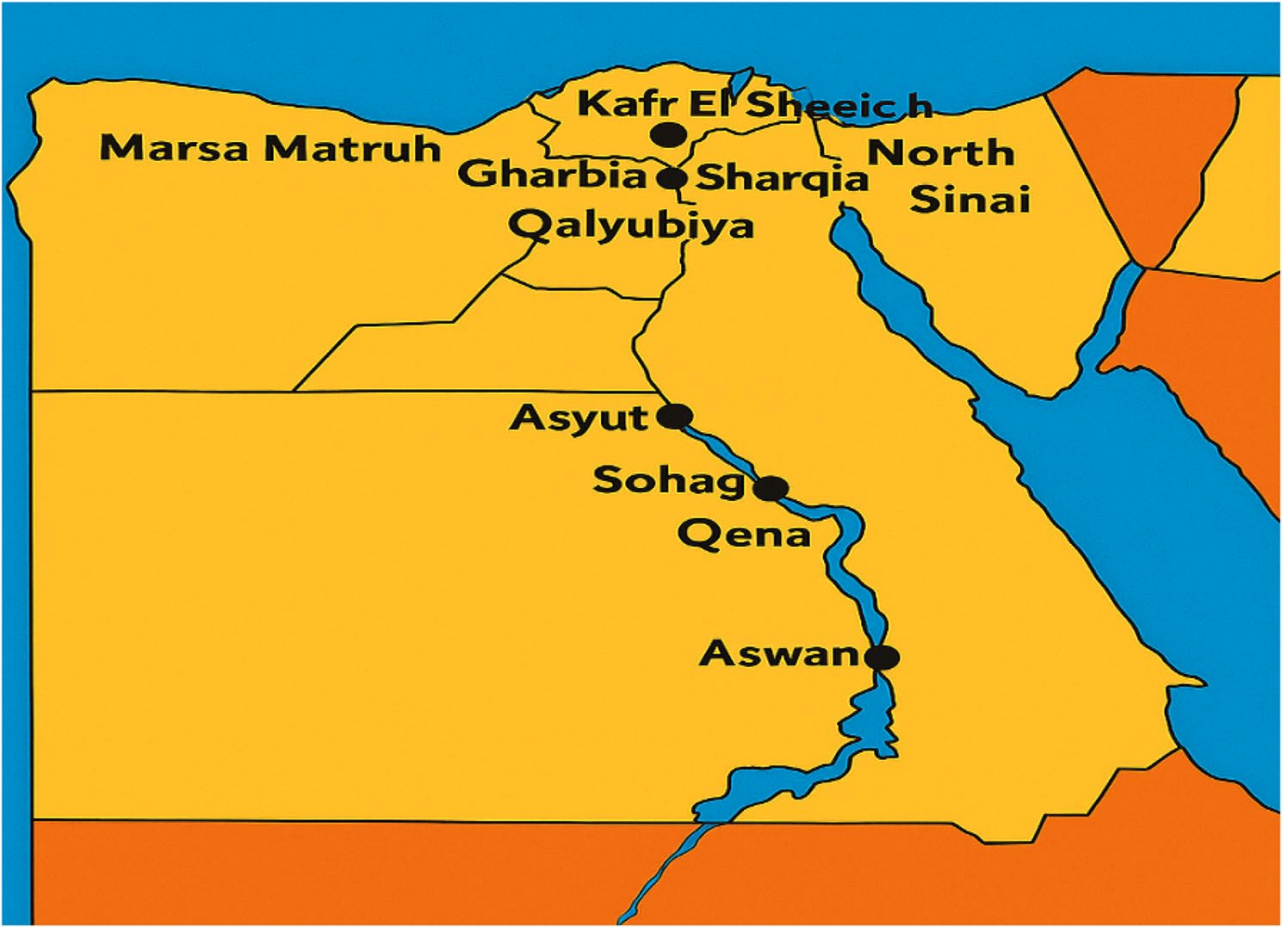
The geographical distribution of the Egyptian provinces where barley landraces were gathered. Adapted from Salam et al. 2025 [26]

This study involved the collection of 81 Egyptian landraces (Table S1) from various agroclimatic zones and Egyptian marketplaces, as shown in Fig. 1. The purpose of the collection was to capture a wide range of genetic diversity in barley adapted to different agro climatic conditions in Egypt. Landraces were sampled based on regional representation, with farmers and local markets providing seeds identified as traditional barley varieties.

### Experimental sites and climatic conditions

This study was conducted at the Bahteem Research Station, Genetic Resources research department, field crop research institution, Agricultural Research Center, Giza, Egypt, on the experimental farm during the primary cropping seasons of 2022 and 2023. The station is located at latitude 30°21′N, longitude 31°13′E, and an altitude of approximately 18 m above sea level. The soil of the experimental site is clay loam in texture and moderately fertile, as established through pre-sowing soil analysis. The mean daily temperature ranged from 15.8 °C to 20.3 °C, with minimum temperatures occasionally dropping to 8.2 °C and the maximum temperatures rising as high as 28.6 °C during later growth stages, with a mean relative humidity of around 62%. The trial was conducted under irrigated conditions with surface irrigation management practices implemented at the appropriate phases of crop development to avoid moisture stress. Rainfall during the growing seasons was minimal and not sufficient to meet crop water requirements; thus, supplemental irrigation was necessary. Normal crop management was followed, including pre-sowing fertilization with 150 kg N/ha and 100 kg/ha calcium superphosphate (15.5% P₂O₅). The experiment was conducted in three replicates using a randomized complete block design (RCBD). Each plot consists of three rows, each 2 m long, with a 20-centimeter space between and within rows. The RCBD was chosen to minimize field variability by accounting for heterogeneity in soil fertility, moisture gradients, and other micro-environmental differences. The plot size was small also (Each plot consists of three rows, each 2 m long, with a 20-centimeter space between and within rows). Also, blocking ensured that treatment comparisons were made within more uniform subgroups. Randomization within blocks was implemented using computer-generated random numbers to eliminate bias and enhance precision.

### The studied characteristics

The following traits were evaluated:

- Phenology traits: days to heading (DH; 10 plants per replicate) and days to maturity (DM; 10 plants per replicate).
- Growth trait: plant height (PH; 10 plants per plot).
- Yield traits: grain yield (GY; entire plot), number of grains per spike (NG/S; 20 spikes per plot), number of spikes per square meter (NS/M²; five 1 m² quadrats per plot), and thousand-grain weight (1000-GW; three samples’ of 1000 grains per plot).

### Statistical analysis

All statistical analyses were performed in R version 4.4.3.

### Principal component analysis (PCA)

Conducted using the prcomp () function from the stats package for base computation, with visualization provided by FactoMineR, factoextra, and ggplot2. PCA was used to investigate the relationships among barley landraces based on agronomic traits.

### Cluster analysis

Euclidean distance matrices were calculated to measure dissimilarities. Ward’s minimum variance method (hclust () in stats) was used for hierarchical clustering, and results were visualized with dendrograms. The optimal number of clusters was determined using the Elbow method, Silhouette analysis, and the Gap statistic, implemented in R (4.4.3). Specifically, the functions fviz_nbclust () from the factoextra package, silhouette () from the cluster package, and clusGap () from the cluster package were used to perform these analyses. Heatmaps were generated using pheatmap and heatmap packages to represent trait variation.

### Correlation analysis

Pearson’s correlation coefficients were calculated using the cor () function in stats. Visualizations were produced using corrplot, pheatmap, and ggcorrplot.

### Path analysis

Conducted in R with the lavaan package (or whichever you used – please confirm). Direct and indirect effects of traits on grain yield were quantified and visualized.

### Genetic parameters

The broad sense heritability was computed as a ratio of the total genotypic variance to phenotypic variance, as stated by Falconer and Mackay [27].

$$\mathrm H^2\;=\;{\mathrm\sigma^2}_{\mathrm g}/{\mathrm\sigma^2}_{\mathrm p}$$

where σ^2^_p_​ represents the phenotypic variance, σ^2^_g_ represents the genotypic variance, and H^2^ stands for heritability in the broadest sense.

The Singh and Chaudhary [28] method was used to evaluate the phenotypic and genotypic coefficients of variation.

$$\mathrm{PCV}\;(\%)\;=\;\left(\mathrm\sigma{p}\;/\;\overline{\mathrm x}\right)\;\times\;100$$

$$\mathrm{GCV}\;(\%)\;=\;\left(\mathrm\sigma{p}\;/\;\overline{\mathrm x}\right)\;\times\;100$$

where x̄ is the grand mean of the characteristic, σp is the phenotypic standard deviation, and σg is the genotypic standard deviation.

At 5% selection intensity (K = 2.06), genetic advance (GA) was computed as follows: $\mathrm{GA}\;=\;\mathrm K\times\mathrm\sigma{p}\times\mathrm H^2$.

$$\mathrm{GAM}\;(\%)\;=\;\left(\mathrm{GA}/\mathrm X^-\right)\;\times\;100$$

where: K represents the selection differential (2.06 at 5%), σp represents the phenotypic standard deviation, H^2^ refers to the broad-sense heritability and the trait mean is represented by X.

## Results

### The agronomic characteristics

**Fig. 2:**
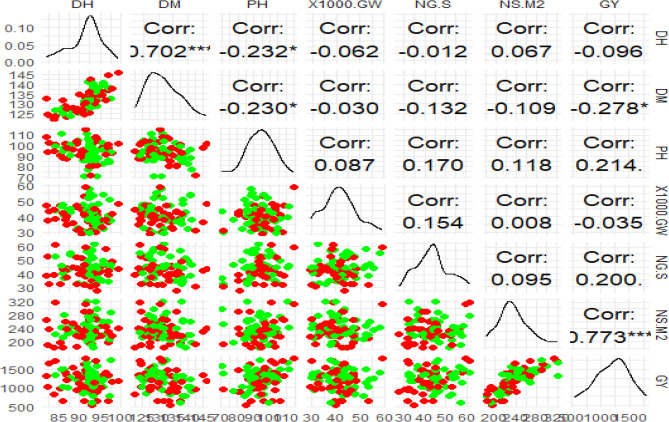
The scatter plot matrix for the pair-wise relationships between the seven variables measured in 81 barley landraces: days to heading (DH), days to maturity (DM), grain yield (GY), number of kernels per spike (NG/S), plant height (PH), number of spikes per square meter (NS/M²), and 1000-grain weight (1000-GW). In the matrix, scatter plots are located in the lower triangle with points alternating between green and red, correlation coefficients are in the upper triangle, and density plots are on the diagonal, which show the distribution of each attribute. The dots are colored in alternating shades of red and green to help with visualization

DH, DM, PH, 1000-GW, NG/S, NS/M^2^, and GY are the seven important traits that are summarized by the pairwise scatter plot matrix of the 81 barley traits (Fig. 2). There was a distinct linear connection in the scatter plot between the days to heading and the days to maturity. The scatter plot between plant height and days to heading showed a modest negative trend. The relationship between plant height and grain yield lacked a discernible pattern (Fig. 2). Grain yield and spike density per square meter showed a positive trend in the scatter plot, with higher yield often corresponding to higher spike density. There was a little upward trend in the scatter plot of the number of grains per spike versus the 1000-grain weight (Fig. 2). Middle heading dates produced larger yields, according to a nonlinear trend in the days to heading vs. grain yield scatter plot. Lower yields were linked to genotypes with exceptionally early or extremely late heading dates (Fig. 2). Most of the features were approximately regularly distributed, according to the density plots along the diagonal. There was a slight right skew in grain yield (Fig. 2).

**Fig. 3:**
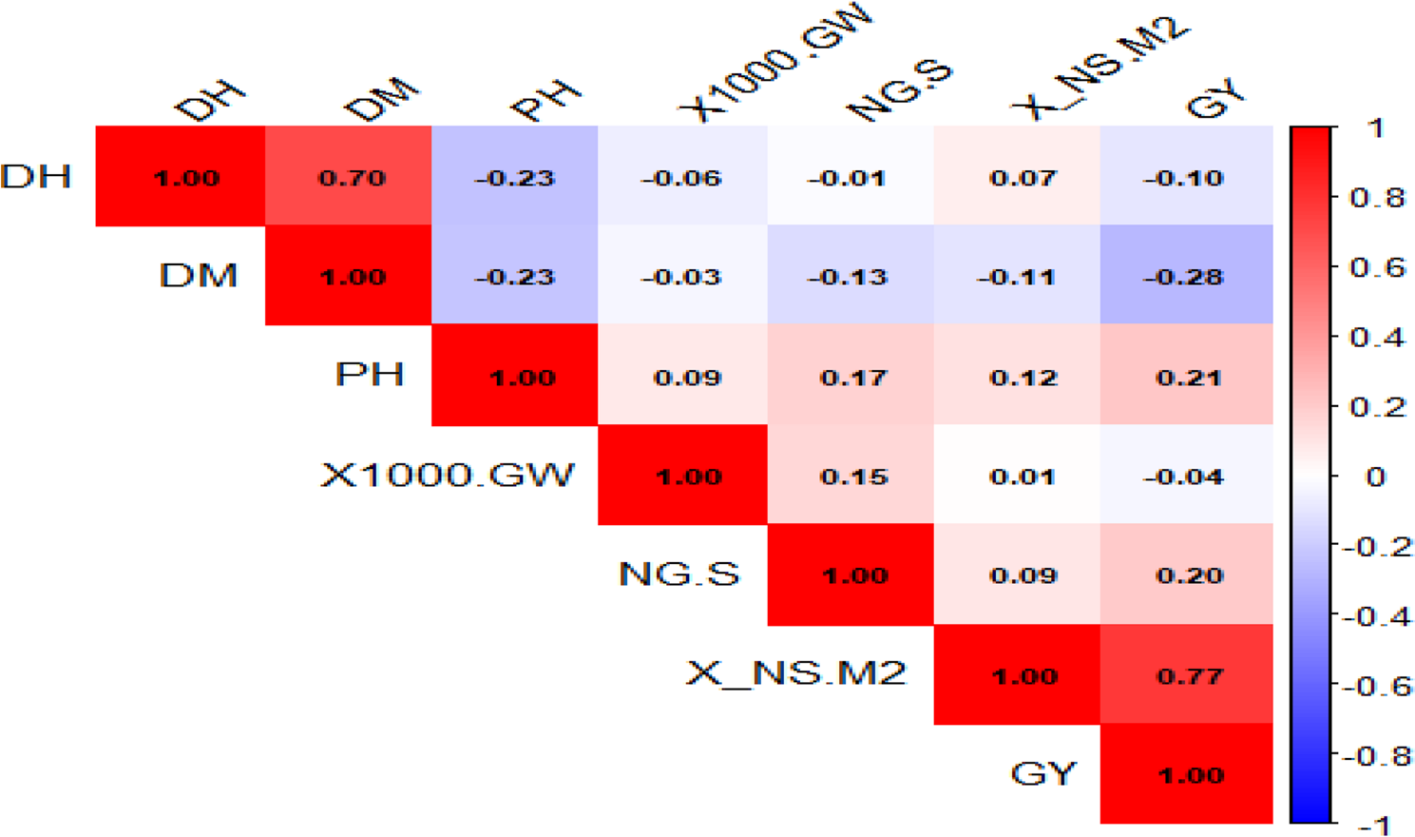
Pearson correlation coefficient heatmap for 81 barley landraces’ agronomic characteristics. The heatmap shows the pair-wise correlations among the traits: days to heading, maturity duration, grain yield, number of grains per spike, plant height, number of spikes per Square Meter, and thousand-grain weight. Correlation coefficients range from − 1 to 1. The red color is used for high positive correlations, blue for high negative correlations, and white for lack of correlation. Hierarchical clustering of the features is displayed to highlight variable associations

The correlation coefficients (Fig. 3) varied from − 0.28 to 0.77. The heatmap showed a number of noteworthy relationships. GY correlations showed a moderately negative connection with NG/S (*r* = −0.40^*^) and a substantial positive correlation with NS/M² (*r* = 0.77^*^). NG/S, 1000-GW, and NS/M² all had very minor positive relationships with PH (*r* = 0.09, 0.17, and 0.12, respectively). The connection between DH and DM was moderate (*r* = 0.70^*^). DH showed extremely weak negative correlations with 1000-GW (*r* = −0.01), NG/S (*r* = −0.06), and NS/M² (*r* = −0.23^*^). Similarly, DM showed very minor associations with 1000-GW (*r* = −0.13), NG/S (*r* = −0.03), and a weak negative connection with NS/M² (*r* = −0.23^*^). 1000-GW had very weak correlations with NG/S (*r* = 0.15) and NS/M² (*r* = 0.01). There was a moderately negative correlation between NS/M² and NG/S (*r* = −0.60^*^) (Fig. 3).

### The genetic parameters for the agronomic characteristics

The genetic parameter estimates for seven agronomic variables in 81 Egyptian barley landraces ranged from 0.97 for DH to 0.99 for the majority of the traits, including PH, 1000-GW, NG/S, NS/M2, and GY (see Table 1). The GCV and PCV for all features were almost equal, indicating a minor environmental influence. GCV was between 3.00% on the days to maturity and 27.74% in GY, while PCV also exhibited almost the same tendency. GY (352.81 g/m²) possessed the highest genetic advance (GA), followed by NS/M^2^ (61.27), while DH and DM had the lowest GA values (Table 1). Genetic progress as a percentage of mean (GAM) was highest for GY (57.45%), then NG/S (30.84%), and 1000-GW (28.61%) (Table 1).

**Table 1:** Estimates of 81 Egyptian barley landraces’ genetic parameters for seven agronomic features

Trait	Heritability (H²)	GCV (%)	PCV (%)	Genetic Advance (GA)	GAM (%)
DH	0.97	3.55	3.6	6.78	7.28
DM	0.98	3	3.03	8.33	6.1
PH	0.99	8.74	8.75	16.8	17.73
1000-GW	0.99	14	14.01	10.47	28.61
NG/S	0.99	14.97	14.97	13.24	30.84
NS/M^2^	0.99	12.52	12.52	61.27	25.82
GY	0.99	27.74	27.74	352.81	57.45

*DH* Days to heading, *DM* Days to maturity, *PH* Plant height, *1000-GW* Thousand grain weight, *NG/S* Number of grains per spike, *NS/M*^2^ Number of spikes per square meter and *GY* Grain yield

### The cluster analysis

**Fig. 4:**
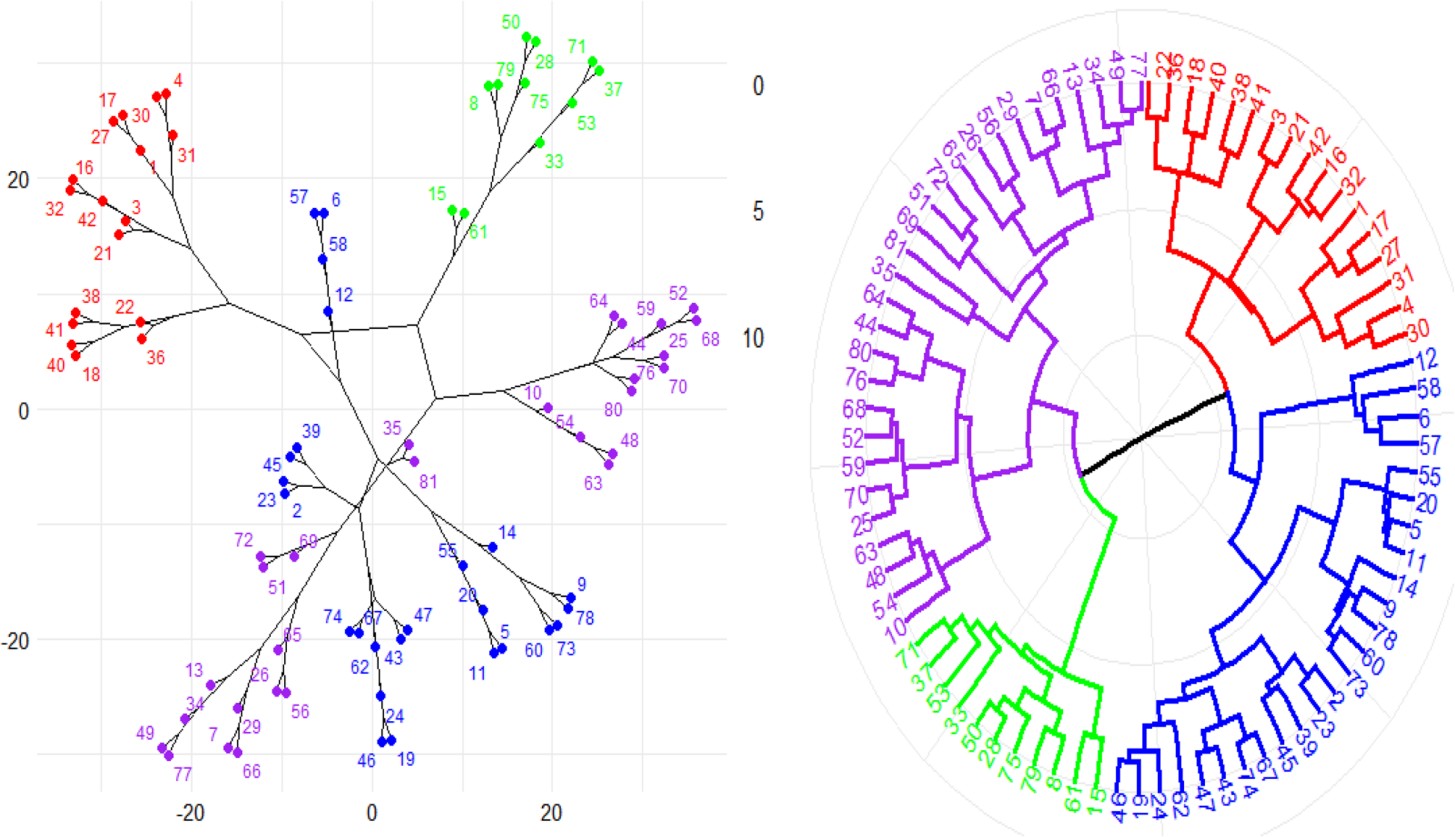
A circular dendrogram displaying 81 barley landraces grouped hierarchically for the agronomic traits. The dendrogram presents four distinct clusters (color-coded) that represent the phenotypic diversity and potential genetic differentiation of the landraces

A panel of 81 barley landraces was subjected to hierarchical clustering for the seven agronomic traits (Fig. 4). Four major clusters were identified in the circular dendrogram, with distinct branch groupings. One cluster consisted of genotypes with relatively intermediate heading and maturity dates but larger thousand-grain weight and grain yield (Fig. 4). The second cluster, on the other hand, had landraces with lower yield components and early phenology. The remaining clusters showed intermediate characteristic combinations that suggested both more differentiated clusters (which suggested increased phenotypic diversity) and compact clusters (which indicated high similarity).

**Fig. 5:**
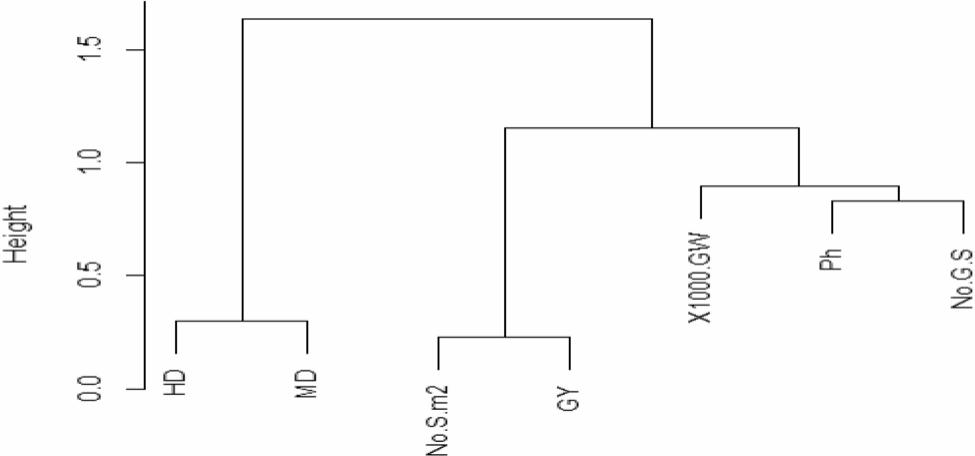
A cluster dendrogram for the seven phenotypic traits (days to heading, maturity duration, grain yield, number of grains per spike, plant height, number of spikes per square meter, and thousand-grain weight of the 81 barley landraces

The hierarchical cluster analysis of the seven traits (DH, DM, PH, 1000-GW, NG/S, NS/M², and GY) revealed disparate grouping based on their correlation (Fig. 5). The following prominent clusters were found in the dendrogram, which shows how the qualities are correlated with one another: The traits were divided into two major groupings using hierarchical clustering. The developmental traits (DH and DM) are included in Cluster I. In contrast, the remaining five characteristics fall into cluster II and are divided into two subgroups (Fig. 5).

Subgroup I contains the NS/M^2^ and GY characteristics. Subcluster II contains PH, NG/S and 1000-GW characteristics (Fig. 5).

### The principal component analysis

**Fig. 6:**
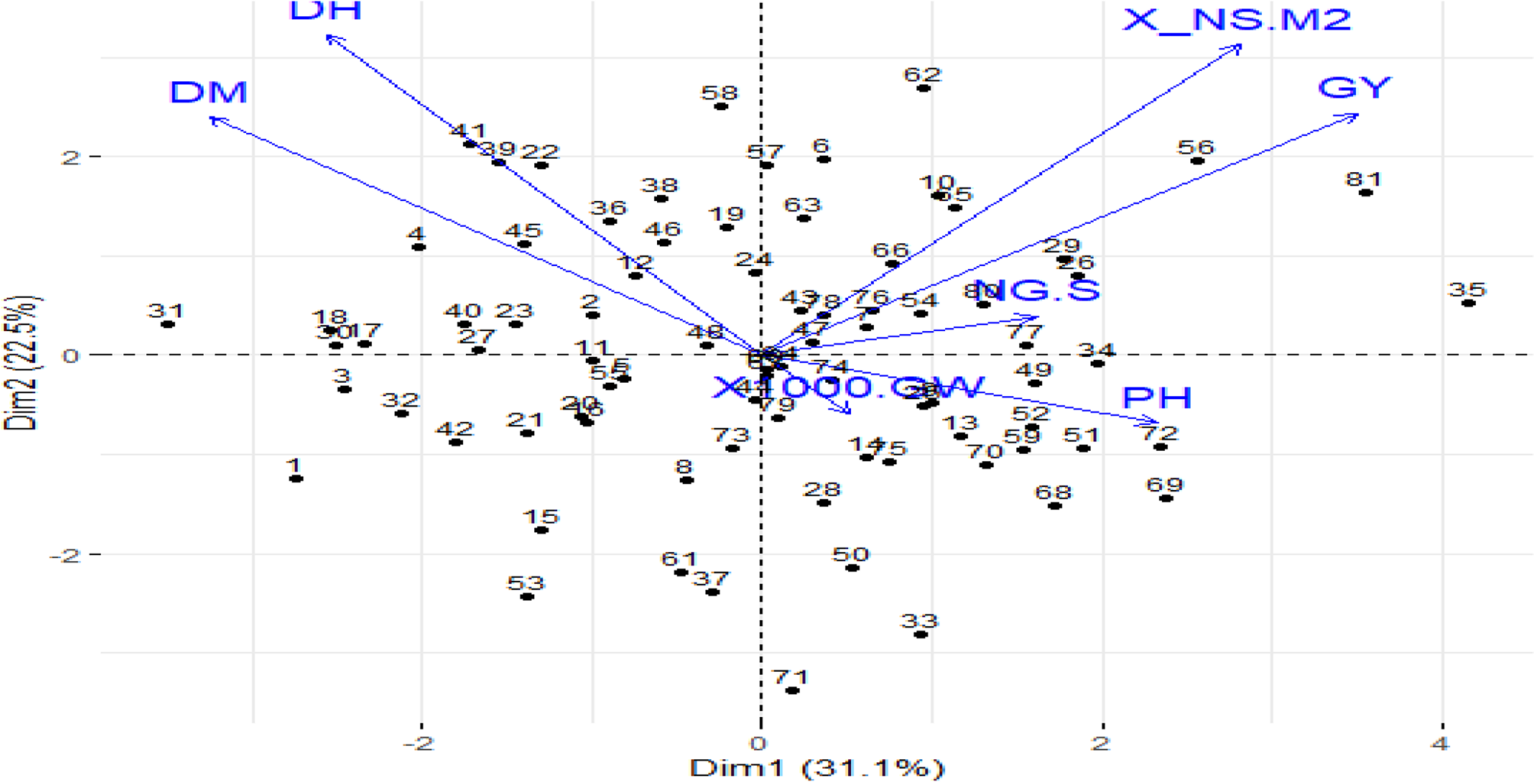
The principal component analysis biplot showing the distribution of 81 barley landraces based on seven agronomic traits: plant height (PH), days to maturity (DM), days to heading (DH), grain yield (GY), number of grains per spike (NG/S), thousand-grain weight (1000-GW), and number of spikes per square meter (NS/M²). Arrows indicate the direction and strength of trait contributions, while numbers represent individual barley landraces

Several features in the biplot contribute considerably to the diversity found among the 81 barley landraces (Fig. 6). The first principal component (Dim1) and the second component (Dim2) are responsible for 31.1% and 22.5% of the total variation, respectively, and 53.6% of the overall variation (Fig. 6).

GY and NS/M^2^ are very strongly positively correlated, as evidenced by the similar vector direction. Landraces near the vectors’ directions, such as landrace 81, both have higher values for these characteristics (Fig. 6). Although they differed slightly from grain weight, PH and 1000-GW were positively correlated. Grain yield had a positive correlation with both DM and DH (Fig. 6). A negative association was observed as the vectors of DM and DH are opposed to the vectors of grain yield (Fig. 6). Landraces 35 and 81 are in the fourth quadrant of Dim1. Landraces 31, 1, and 53 are on the opposite side of the quadrant. Middle landraces near the origin (e.g., landrace 44) exhibit normal performance on most of the traits (Fig. 6).

Principal Component 1 (PC1) explained the most variation among the 81 barley landraces, with attributes loaded positively and negatively. PC1 showed the largest positive loadings for GY (0.518) and NS/M² (0.416) (Table 2). In contrast, DH (−0.379) and DM (−0.482) had negative loadings (Table 2). Principal Component 2 (PC2) showed a different pattern of trait relationships. While PH exhibited a minor positive loading (0.119), other features like DH (−0.563), NS/M² (−0.546), GY (−0.425), and DM (−0.417) had moderate to high negative loadings (Table 2). With PC3, NG/S (−0.594) and 1000-GW (−0.697) predominate unfavorably, and PH (−0.242) loads negatively as well (Table 2).

**Table 2:** Seven characters from 81 bread wheat genotypes were analyzed using the first three main components (PCs)

Trait	PC1	PC2	PC3
DH	−0.379	−0.563	−0.186
DM	−0.482	−0.417	−0.183
PH	0.344	0.119	−0.242
1000-GW	0.076	0.101	−0.697
NG/S	0.238	−0.068	−0.594
NS/M^2^	0.416	−0.546	0.123
GY	0.518	−0.425	0.130

*DH *Days to heading, *DM* Days to maturity, *PH* Plant height, *TGW* Thousand grain weight, *NG/S* Number of grains per spike, *NS/M*^2^ Number of spikes per square meter and *GY* Grain yield

**Fig. 7:**
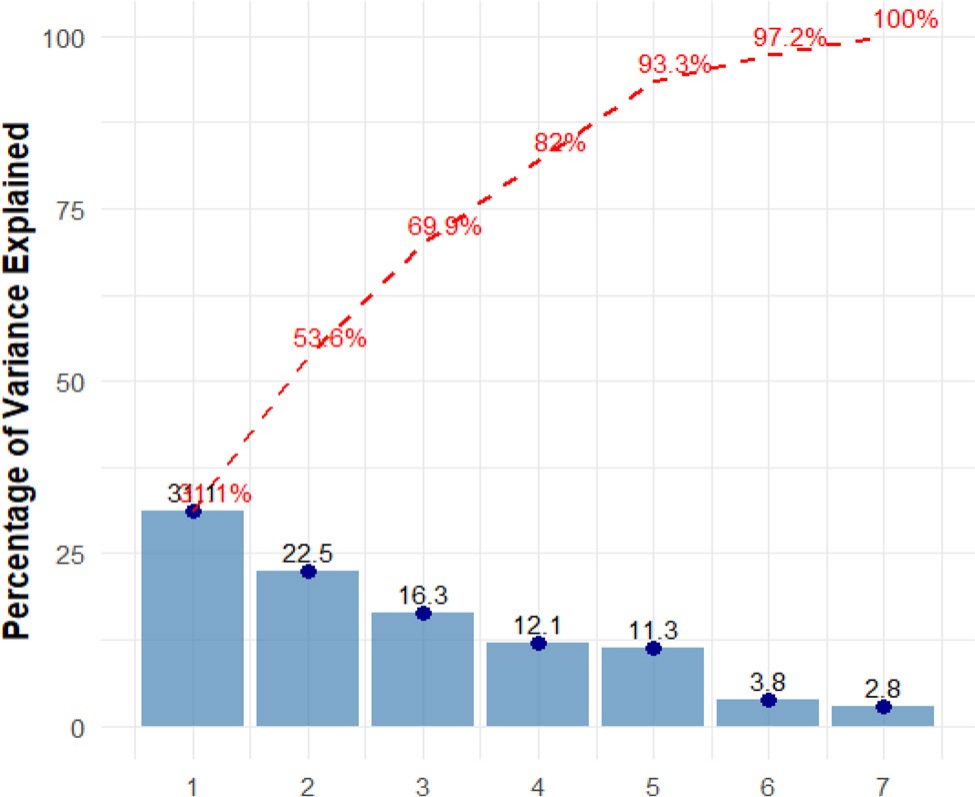
A scree plot illustrating the percentage of variance for 81 barleys that can be accounted for by principal components. Each PC is highlighted with points, and the variance explained is displayed as bars. The cumulative variance is shown by the red dashed line. The plot's cumulative variance and precise variance percentages are shown in the annotations

Related to the scree plot, we can note that the first principal component explains a substantially higher amount of variance compared to the subsequent components (Fig. 7). We can see a rapid reduction in variance explained by PC1, followed by a moderate decline as we proceed along the principal components. The early components explain the majority of the variance, with the higher components contributing less and less progressively (Fig. 7). The curve shows a noticeable inflection point, or “elbow,” between PC2 and PC3, where the rate of variance explained begins to slow significantly. This means that the first two or three components capture most of the meaningful variance, while additional components contribute marginally (Fig. 7).

### The path analysis

**Fig. 8:**
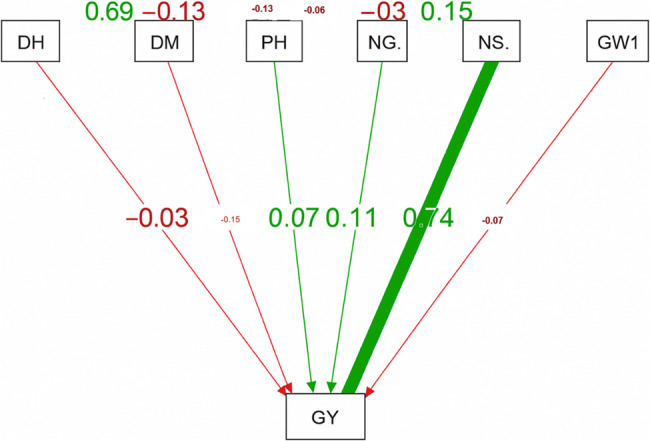
The direct and indirect effects of days to heading, days to maturity, plant height, number of grains per spike, number of spikes per square meter and 1000-grain weight characteristics on grain yield. The red lines represent the negative effect, whereas the green lines represent the positive effect

The path analysis identified the agronomic traits’ direct and indirect effects on the 81 barley landraces’ grain yield (Fig. 8). NS/M^2^ significantly increased grain yield directly (0.74). Grain yield was directly impacted by NG/S and PH in modest but substantial ways (coefficients of 0.11 and 0.07, respectively). Grain yield was directly impacted negatively by DM and DH. Concerning the indirect effects, DH affected GY via PH (−0.13) and DM (−0.23). GY was negatively impacted by DM indirectly through NG/S (−0.06) and NS/M^2^ (−0.09). Because taller plants tended to set more grains per spike, they indirectly contributed positively to GY through NG/S (0.17). Additionally, NS/M^2^ indirectly affected GY through 1000-GW (0.15) and NG/S (0.12) (Fig. 8).

## Discussion

This study examined the genetic diversity and interrelationships among agronomic traits in 81 Egyptian barley landraces using multivariate statistical analyses. The findings provide important insights for barley improvement programs focused on yield and adaptation.

### Trait relationships and correlations

The scatter plot matrix reveals a wide range of distributions and patterns for the eight traits tested in the 81 barley landraces (Fig. 2). Notably, most of the characteristics do not have strong linear correlations with one another, suggesting that the traits are influenced by independent variables or interacting factors [29–31]. The pairwise scatter plot matrix provided insightful information regarding interactions between the seven barley traits and a graphical foundation for interpreting the yield and related traits’ genetic and physiological basis [32, 33]. In our outputs, there was a distinct linear connection in the scatter plot between the DH and DM, demonstrating a close relationship between these qualities throughout development. This interaction emphasizes the importance of early heading selection in order to achieve early maturity, which will prove valuable under terminal drought or heat stress conditions [34]. There was no clear trend in the link between PH (cm) and GY (Ton/ha). A modest number of high-yielding genotypes were identified in short plants, though, which would indicate that reduced PH has a possible benefit in terms of yield maximization [35].

The negative weak association between PH and DH suggests that there may be a trade-off between vegetative growth and the timing of reproductive development [5, 13, 36]. This finding supports the concept of “dwarfing genes” in cereal crops, where plants with shorter heights have superior resource allocation and higher yields. The nonlinear relationship between DH and GY suggests that intermediate heading dates may be best for achieving maximum yield [5]. Very early or very late heading genotypes yielded less, possibly because they were not best suited to the environmental conditions. This is an significant result for breeding programs, as it suggests that selection for intermediate heading dates can optimize yield potential [37].

The density plots (Fig. 2) demonstrated that most of the traits were normally distributed, meaning that the studied population of barley was genetically variable [5]. The modest right skew of grain yield points to the presence of high-yielding genotypes that would be valuable for breeding. These landraces would be excellent donors for increasing yield in elite breeding lines. These scattered distributions highlight the need for further studies on the genetic and environmental components of yield [38]. The pairwise scatter plot matrix could depict the complex relationships between barley traits, providing a foundation for future genetic and breeding studies [39]. A slight right skew in grain yield suggested that there were a few high-yielding genotypes that would be valuable to employ in breeding initiatives [40]. Further investigations into the genetic nature of correlations between the qualities could be performed in future study with the utilization of genomic techniques, allowing the production of superior barley varieties with perfect combinations of traits [41].

The analysis of correlation helps elucidate the relationship between agronomic characters within barley landraces that can be utilized to inform breeding and selection [42]. The significant positive correlation between days to heading and days to maturity reflects the very close association between heading date and maturity in barley [43]. This is what might be expected on a physiological basis, with earlier heading leading to earlier maturity. This implies that breeders aiming for early-maturing varieties should consider both traits together due to their interdependence. The heatmap of correlations confirmed the significant positive association between spikes per square meter (NS/M²) and grain yield (*r* = 0.77), highlighting spike density as a critical yield determinant. However, the positive association (*r* = 0.17) between plant height and the number of grains per spike was weak and statistically insignificant, so any inference that taller plants produce more grains per spike should be made with caution [44]. The overall weak positive tendency (*r* = 0.15) between 1000-grain weight and the number of grains per spike suggests a slight trade-off, where genotypes with larger grains may have slightly fewer grains per spike [45]. However, the correlation is weak, indicating that improving grain size may not severely compromise grain number per spike [46]. These identified correlations can guide trait prioritization in breeding programs by outlining the need to consider both direct and indirect effects of selection [45, 46]. These findings provide breeders with insights on which traits to prioritize depending on breeding goals [41].

### The genetic parameters and selection potential

The High heritability estimates and narrow differences between genotypic and phenotypic coefficients of variation for yield components indicate predominantly additive genetic control and minimal environmental influence, indicating these traits can be effectively selected in early generations [47]. Particularly, grain yield, NS/M², NG/S, and 1000-GW demonstrated high genetic advance as a percentage of the mean (GAM), reinforcing their breeding value [5, 41]. Conversely, lower heritability and GAM for DH and DM reflect less genetic variability, suggesting limited improvement potential through direct selection [48].

### Cluster groupings and landrace adaptation

The circular cluster analysis (Fig. 4) nicely distinguished four unique groups within the barley landraces and demonstrated substantial phenotypic heterogeneity, implying hidden genetic variability [49]. The mode of clustering was dominated by variation in the major agronomic traits, such as DH, DM, and GY(Ton/ha), which are critical for barley adaptation to diverse environmental conditions, as well as maximum yield performance optimization [50].

Furthermore, utilizing a circular dendrogram made it easier to visualize hierarchical structure in a large data set without creating a lot of visual distraction and enhanced the interpretability of complex clustering patterns [51]. The distinct clusters identified can form the basis of the choice of parental lines for crossing to maximize genetic diversity and heterosis potential [27]. Furthermore, the integration of these phenotypic clusters with molecular marker analysis in follow-up studies may provide additional information regarding the genetic architecture of these agronomic traits [52, 53]. Multi-environment trials would also be valuable to confirm the stability of the clusters as well as test for genotype-by-environment interactions [54, 55]. The advanced circular cluster analysis highlights how significant it is to use sophisticated clustering and visualization methods when looking at intricate trait variation [56]. Not only does the method enhance our understanding of phenotypic diversity in barley landraces, but it also provides a strategic framework for the improvement of barley through targeted breeding interventions [57].

### Cluster differentiation and characteristics contributions

The use of standardization (Fig. 5) ensured that each character contributed similarly to the analysis, allowing the clustering algorithm to split the landraces based on actual performance differences [51]. The cluster analysis provides an understanding of the relationship among the studied traits, their interdependence, and potential functions in the performance and yield of barley [21].

Characters like DH, DM, and GY (Ton/ha) were identified to be the major drivers of cluster formation (Fig. 5). For instance, landraces belonging to a specific cluster may possess intermediate heading and maturity dates accompanied by increased GY and larger 1000-GY, which are signals of their employability in conditions where early establishment and high yield are necessary [55, 58]. Clusters with shorter phenology or reduced yield indicate other adaptation strategies that would be desirable for breeding under certain agronomic conditions [14]. The grouping of HD and DM together indicates a strong correlation between heading and maturity dates, most likely because both traits relate to the phenological development of barley [48]. Both these traits are determined by shared environmental and genetic factors and thus fall under the breeding selection criterion for being interdependent [59–61]. Cluster 2, which contains 1000-GW, NG/S, and NS/M², shows their common contribution towards grain yield. These traits are crucial for the determination of yield potential as they explain the number of grains and their respective weights [23]. The close correlation among these traits (Fig. 5) suggests that breeding activities for any one of these traits may have the indirect effect of enhancing the others and thus the yield [28]. Grain yield characteristics and the NS/M^2^ are included in one subgroup 1, which implies that an increase in spikes per unit area contributes substantially to grain yield [60]. Also, the classification of PH with 1000-GW and NG/S in one sub-cluster suggests that PH has the potential to influence grain size as well as the NG/S [62]. They cumulatively influence ultimate grain yield and are factors of importance in the selection of high-yielding and lodging-resistant barley varieties [63].

### PCA interpretation

The PCA biplot provides considerable information about the correlations between characteristics and landrace performance, allowing selection for barley improvement initiatives [64]. The PCA biplot shows that some groups of barley landraces have similar profiles of characteristics, possibly due to common genetic background, environmental acclimatization, or breeding history [65]. In the current analysis of 81 barley accessions, the PCA biplot (Fig. 6) clearly illustrates distinct groupings, such as accessions 56, 81, and 35 that are positioned far from the origin, implying unique performance profiles, likely driven by higher values for grain yield, number of spikes per square metre, and associated traits. Also, in our findings, the high correlation between GY and NS/M^2^ suggests that increased spike density is critical for yield improvement. However, 1000-GW is not highly correlated with GY, which indicates that heavier grains do not necessarily translate into increased total yield in this data set [65]. This suggests that yield breeders should focus on spike density and grain number over grain weight alone [61]. The inverse association between GY and maturity traits (DM, DH) indicates that there is a greater tendency for early-maturing landraces to produce more than late-maturing landraces. This may be due to them being capable of completing their growth cycle before adverse environmental situations, such as drought or heat stress, get a chance to influence grain filling [61]. This becomes crucial for breeding programs aimed at enhancing adaptation for short growing seasons [47]. Our results also demonstrated that although plant height and 1000-GW differ significantly from grain weight, they have a positive correlation, suggesting that grain yield is more influenced by the NS/M2 and the number of grains per spike than by 1000-GW alone [66].

The result of the PCA loadings provides valuable information regarding how the agronomic traits of the 81 barley landraces contribute to the overall variation in the dataset [66]. The high positive loadings of NS/M2 and GY on PC1 indicate that high-yielding landraces produce more seeds per unit area, a key characteristic for productivity [64]. Early-maturing varieties may optimize resource allocation to seed production rather than extended vegetative growth, according to the negative loadings of DH and DM [63]. This is in line with evidence showing that early maturity can enhance yield stability under stressful conditions (e.g., in drought-prone environments) [67]. Negative DH/DM vs. NS/M2/GY correlations in PC2 show that maturation delay reduces seed number and yield. This could be the result of resource rivalry between prolonged vegetative growth and reproduction [61]. Low positive PH loading suggests that tall plants prefer maturity delays to some degree, but this relationship is weaker [60, 68]. PC3 also detects a classic agronomic trade-off: landraces with larger seeds (1000-GW) and fewer grains per spike (NG/S) have shorter PH, cm. This suggests that larger-seeded plants may allocate more resources to seed growth at the expense of vegetative development [63]. This trade-off is significant for particular environment-specific breeding programs because larger seeds may increase seedling vigor but reduce total seed number [63].

The scree plot demonstrates that a limited number of main components can account for the majority of the variability in the data set based on the rapid decrease in variance for the first few components, followed by a more continuous fall [6, 61, 69]. PC1 accounts for the largest proportion of variance, implying that it outlines the most significant patterns in the data, perhaps representing the combined effect of several traits [70]. PC2 also accounts for a high proportion of variance, contributing additional information on landrace trait differentiation.

### The path analysis

The results of path analysis are highly informative concerning the relative contribution of different traits to GY (Ton/ha) in barley landraces [71]. Regarding the direct influence, the significant direct effect of spikes’ number per square meter on GY underlines its relevance as a major constituent of yield [72]. Our results agree with previous studies on barley breeding, placing plant architecture (NS/M^2^) at the top of the determining factors of grain yield [71, 73]. The high-valued positive path from NG/S to GY highlights the importance of grain quantity per spike as a selection feature in barley breeding efforts [71]. Furthermore, the indirect relationship between DH and DM and grain yield indicates that being early will be advantageous if it is linked to a high grain-setting ability [74]. This confirms the breeding strategy of selecting genotypes with an optimum early flowering and sufficient grain-filling period for attaining maximum yield [73]. Remarkably, GY was somewhat impacted negatively by 1000-GW (−0.07), indicating a little trade-off between grain weight and total yield [70]. Breeders should instead focus on traits that enhance spike density and grain number to realize maximum overall yield potential [75].

In these findings, PH had a substantial direct effect on GY, showing that taller plants contributed to enhanced yield [71]. Additional indirect benefits of the number of spikes per square meter were obtained by the number of grains per spike (0.12) and 1000-GW (0.15), which increased its beneficial yield contribution [69]. The 1000-GWh had limited indirect effects, indicating its principal contribution comes from direct actions [68].

### The implications for breeding programs

The biplot demonstrated that breeding methods for high grain yield should include the selection of landraces 35 and 81, which fall along yield-related characteristics. Landraces 1 and 53, which are connected to late maturity, would be better suited for environments that benefit from a longer growing season. Also, the moderate correlation between PH and 1000-GW suggests that optimization of plant height without excessive increase in grain weight could improve lodging resistance without productivity loss [71, 76]. It can be used to tell breeders whether to select landraces based on certain traits, such as grain yield or grain weight, according to breeding aims [77]. Biplot results indicated that early-maturing landraces will be more productive in the target environment due to the negative connection between GY and maturity parameters (DM and DH). Late-maturing landraces, such as 1 and 53, are not suitable for places with terminal drought stress or shorter growing seasons [71]. Late-maturing genotypes would be helpful in conditions with longer growth seasons and adequate moisture availability [61]. The visualization also reveals that some landraces may help in enhancing plant height or spike length-related features, which would be beneficial for the adaptation of barley varieties to varied climates or farming settings [72]. The PCA biplot is a notable display of the 81 barley landraces’ variation and indicates key characteristics underlying this variation [52]. These findings can be employed to inform future breeding and finding landrace-specific traits of possible agricultural value [67].

Selection of landraces with large PC1 scores may emphasize yield and early flowering [73]. Landraces with moderate PC2 scores could balance development timing and architecture [74]. Seed size-based breeding programs (e.g., for drought tolerance) need to track trade-offs with grain number and height [76].

The cluster analysis results showed that early or late heading/maturity might be chosen at the same time because they are so near. The greatest approach to increasing grain output would be to focus on NS/M^2^. Traits like PH, 1000-GW, and NG/S may have an inter-relationship affecting one another, hence balanced selection is required [75, 76].

### Study limitations and field challenges

Despite the useful insights provided, this study has limitations. Analysis in a single location limits generalization across several situations. Another limitation is the lack of molecular or genomic characterization in this investigation. While we focused on phenotypic and agronomic variability, using SNP markers, genome-wide association studies (GWAS), or other omics techniques would allow for a more comprehensive knowledge of the landraces’ genetic architecture and breeding potential. Furthermore, despite the huge sample size, it is unlikely to cover the entire range of barley genetic diversity in Egypt or elsewhere. Future multi-location investigations using molecular marker analysis are required to validate these findings and improve breeding strategies.

Several logistical obstacles arose during this experiment, including a lack of seeds and potential landrace impurity complications, necessitating meticulous sampling to ensure representation. Furthermore, data collection was hampered by labour and time availability, particularly at peak phenological stages, which may have introduced variability [78]. There were additional environmental variations over the growth season, such as irregularity in rainfall distribution, which most likely affected trait expression and experimental homogeneity. Integrating molecular markers and genomic tools to these agronomic findings would improve the robustness of trait selection, offering a stronger genetic basis for breeding decisions.

## Conclusion

Related to the correlation analysis, the strongest positive association was found between the number of spikes per square meter and grain yield, indicating that it may be a significant factor in increasing yield. There was a reasonably positive association between days to heading and days to maturity, indicating that phenological development occurs concurrently. The cluster analysis indicated substantial characteristic groupings, highlighting their interconnectedness and probable involvement in barley performance. The findings highlight the necessity of taking trait dependency into account when developing breeding strategies, and they show that focusing on specific sets of features may result in improved yield and adaptation. The pathway analysis gives useful insights for barley breeding initiatives aimed at improving yield. To increase productivity, breeding programs should focus on improving spike density and grains per spike, as NS/M^2^ and NG/S were found to be the main drivers of yield. The PCA biplot also detects significant agronomic trade-offs, particularly between high yield and early maturity. The findings suggest that early maturity and spike density must be accorded prominence in breeding programs to enhance productivity. The principal component analysis results can be used to guide marker-assisted selection or genomic breeding schemes to plan favorable sets of traits in landraces to meet specific agro-ecological conditions. Breeders can directly apply these insights by focusing marker-assisted or genomic selection on these traits to develop high-yielding, early-maturing cultivars tailored to specific growing conditions. Future studies can explore causal connections among such characteristics and how they remain stable across different settings to further maximize breeding strategies. However, a limitation of this study is that it was conducted in a single environmental setting, which may affect the generalizability of the findings. Overall, the most important finding is that grains per spike (NG/S) and spike density (NS/M^2^) are yield-determining factors, and should be given top priority in selection programs. These findings are significant for overall agricultural improvement, especially in addressing food security and in making barley production more suitable to climate change.

## Supplementary Information

Supplementary Material 1.

## Data Availability

Not applicable.
