# Supplementary material for: Multivariate analysis of agronomic characteristics in some Egyptian barley landraces: a field-based quantitative study
Source: BMC Plant Biol. 2025 Nov 14;25:1565. doi: 10.1186/s12870-025-07512-8 (PMC12616932; doi:10.1186/s12870-025-07512-8)
Supplement: Supplementary file 1 — Supplementary Material 1. [file 12870_2025_7512_MOESM1_ESM.docx]

**Table S1** The origin and obtained location of the studied 81 barley landraces

| Genotypes | Origin | Location |  |
| --- | --- | --- | --- |
| G1 | Km N of Idfu by El Sabaya village 35 | Qena, Egypt | |
| G2 | Km N of Idfu by El Sabaya village 35 | Qena, Egypt | |
| G3 | Km N of Idfu by El Sabaya village 35 | Qena, Egypt | |
| G4 | Km N of Idfu by El Sabaya village 35 | Qena, Egypt | |
| G5 | Km N of Idfu by El Sabaya village 35 | Qena, Egypt | |
| G6 | Km N of Idfu by El Sabaya village 35 | Qena, Egypt | |
| G7 | Beni feez - Near Sidfa | Assiut, Egypt | |
| G8 | Beni feez - Near Sidfa | Assiut, Egypt | |
| G9 | Beni feez- Near Sidfa | Assiut, Egypt | |
| G10 | Beni feez- Near Sidfa | Assiut, Egypt | |
| G11 | Beni feez- Near Sidfa | Assiut, Egypt | |
| G12 | Beni feez- Near Sidfa | Assiut, Egypt | |
| G13 | Beni feez - Near Sidfa | Assiut, Egypt | |
| G14 | Beni feez - Near Sidfa | Assiut, Egypt | |
| G15 | Beni feez - Near Sidfa | Assiut, Egypt | |
| G16 | Beni feez - Near Sidfa | Assiut, Egypt | |
| G17 | Armant local market | Qena, Egypt | |
| G18 | Armant local market | Qena, Egypt | |
| G19 | Armant local market | Qena, Egypt | |
| G20 | Armant local market | Qena, Egypt | |
| G21 | Armant local market | Qena, Egypt | |
| G22 | Armant local market | Qena, Egypt | |
| G23 | Armant local market | Qena, Egypt | |
| G24 | Armant local market | Qena, Egypt | |
| G25 | Armant local market | Qena, Egypt | |
| G26 | Armant local market | Qena, Egypt | |
| G27 | 4 Km W of Qena on the road to Dandara temple | Qena, Egypt | |
| G28 | 5 Km W of Qena on the road to Dandara temple | Qena, Egypt | |
| G29 | 6 Km W of Qena on the road to Dandara temple | Qena, Egypt | |
| G30 | 7 Km W of Qena on the road to Dandara temple | Qena, Egypt | |
| G31 | 8 Km W of Qena on the road to Dandara temple | Qena, Egypt | |
| G32 | 9 Km W of Qena on the road to Dandara temple | Qena, Egypt | |
| G33 | 10 Km W of Qena on the road to Dandara temple | Qena, Egypt | |
| G34 | 11 Km W of Qena on the road to Dandara temple | Qena, Egypt | |
| G35 | 12 Km W of Qena on the road to Dandara temple | Qena, Egypt | |
| G36 | 13 Km W of Qena on the road to Dandara temple | Qena, Egypt | |
| G37 | 14 Km W of Qena on the road to Dandara temple | Qena, Egypt | |
| G38 | 15 Km W of Qena on the road to Dandara temple | Qena, Egypt | |
| G39 | 16 Km W of Qena on the road to Dandara temple | Qena, Egypt | |
| G40 | 17 Km W of Qena on the road to Dandara temple | Qena, Egypt | |

**Table S1** Continued

| Genotypes | Origin | Location |
| --- | --- | --- |
| G41 | Ben - Rafi | Assiut, Egypt |
| G42 | Ben - Rafi | Assiut, Egypt |
| G43 | Ben - Rafi | Assiut, Egypt |
| G44 | Ben - Rafi | Assiut, Egypt |
| G45 | Ben - Rafi | Assiut, Egypt |
| G46 | Qena | Qena, Egypt |
| G47 | Qena | Qena, Egypt |
| G48 | Qena | Qena, Egypt |
| G49 | Qena | Qena, Egypt |
| G50 | Qena | Qena, Egypt |
| G51 | El Balyana | Sohag, Egypt |
| G52 | El Balyana | Sohag, Egypt |
| G53 | El Balyana | Sohag, Egypt |
| G54 | El Balyana | Sohag, Egypt |
| G55 | El Ma sara-Dakhla | New valley, Egypt |
| G56 | El Ma sara-Dakhla | New valley, Egypt |
| G57 | El Ma sara-Dakhla | New valley, Egypt |
| G58 | El Ma sara-Dakhla | New valley, Egypt |
| G59 | 3 Km S of Beni Mazar | Minia, Egypt |
| G60 | 3 Km S of Beni Mazar | Minia, Egypt |
| G61 | 3 Km S of Beni Mazar | Minia, Egypt |
| G62 | 3 Km S of Beni Mazar | Minia, Egypt |
| G63 | 3 Km S of Beni Mazar | Minia, Egypt |
| G64 | 3 Km S of Beni Mazar | Minia, Egypt |
| G65 | 11 Km S of Ihnasya El Madina | Benisuef, Egypt |
| G66 | 11 Km S of Ihnasya El Madina | Benisuef, Egypt |
| G67 | 11 Km S of Ihnasya El Madina | Benisuef, Egypt |
| G68 | 11 Km S of Ihnasya El Madina | Benisuef, Egypt |
| G69 | 11 Km S of Ihnasya El Madina | Benisuef, Egypt |
| G70 | 3 Km of El Fayoum (El Karadisa) | Fayoum, Egypt |
| G71 | 3 Km of El Fayoum (El Karadisa) | Fayoum, Egypt |
| G72 | 3 Km of El Fayoum (El Karadisa) | Fayoum, Egypt |
| G73 | 3 Km of El Fayoum (El Karadisa) | Fayoum, Egypt |
| G74 | 3 Km of El Fayoum (El Karadisa) | Fayoum, Egypt |
| G75 | 3 Km of El Fayoum (El Karadisa) | Fayoum, Egypt |
| G76 | 3 Km of El Fayoum (El Karadisa) | Fayoum, Egypt |
| G77 | Giza | Giza, Egypt |
| G78 | Giza | Giza, Egypt |
| G79 | Giza | Giza, Egypt |
| G80 | Giza | Giza, Egypt |
| G81 | Giza | Giza, Egypt |
